# Alzheimer’s disease manifests abnormal sphingolipid metabolism

**DOI:** 10.3389/fnagi.2024.1368839

**Published:** 2024-05-07

**Authors:** Baasanjav Uranbileg, Hideaki Isago, Eri Sakai, Masayuki Kubota, Yuko Saito, Makoto Kurano

**Affiliations:** ^1^Department of Clinical Laboratory Medicine, Graduate School of Medicine, The University of Tokyo, Tokyo, Japan; ^2^Nihon Waters K.K., Tokyo, Japan; ^3^Tokyo Metropolitan Geriatric Hospital and Institute of Gerontology, Tokyo, Japan

**Keywords:** Alzheimer’s disease (AD), sphingosine (Sph), ceramide 1-phosphate (Cer1P), ceramide (Cer), sphingomyelin (SM)

## Abstract

**Introduction:**

Alzheimer’s disease (AD) is associated with disturbed metabolism, prompting investigations into specific metabolic pathways that may contribute to its pathogenesis and pathology. Sphingolipids have garnered attention due to their known physiological impact on various diseases.

**Methods:**

We conducted comprehensive profiling of sphingolipids to understand their possible role in AD. Sphingolipid levels were measured in AD brains, Cerad score B brains, and controls, as well as in induced pluripotent stem (iPS) cells (AD, PS, and control), using liquid chromatography mass spectrometry.

**Results:**

AD brains exhibited higher levels of sphingosine (Sph), total ceramide 1-phosphate (Cer1P), and total ceramide (Cer) compared to control and Cerad-B brains. Deoxy-ceramide (Deoxy-Cer) was elevated in Cerad-B and AD brains compared to controls, with increased sphingomyelin (SM) levels exclusively in Cerad-B brains. Analysis of cell lysates revealed elevated dihydroceramide (dhSph), total Cer1P, and total SM in AD and PS cells versus controls. Multivariate analysis highlighted the relevance of Sph, Cer, Cer1P, and SM in AD pathology. Machine learning identified Sph, Cer, and Cer1P as key contributors to AD.

**Discussion:**

Our findings suggest the potential importance of Sph, Cer1P, Cer, and SM in the context of AD pathology. This underscores the significance of sphingolipid metabolism in understanding and potentially targeting mechanisms underlying AD.

## 1 Introduction

Alzheimer’s disease (AD) is a widespread neurodegenerative condition that affects millions of individuals worldwide, with a far-reaching impact on not only the patients themselves, but also their families, society, and healthcare systems (Winblad et al., 2016). AD is responsible for approximately two-thirds of all dementia cases (Livingston et al., 2017) and is characterized by the presence of aggregates of pathologically misfolded proteins, mainly of amyloid β (Aβ), which is a product of the proteolytic cleavage of the transmembrane Aβ precursor protein (Karran et al., 2011; Brunkhorst et al., 2014). The precise pathogenesis of AD remains incompletely understood, and the true significance of the numerous disruptions observed in these patients remains ambiguous. Several established mechanisms have been linked to neuronal and synaptic degeneration in the brains of patients with AD. These established mechanisms include oxidative damage, perturbed redox signaling, and mitochondrial dysfunction (Chen and Zhong, 2014; Yuyama et al., 2014; Ashleigh et al., 2023), impaired glucose metabolism (Serviddio et al., 2011), and an inflammatory response (Hansen et al., 2018; Singh, 2022).

However, the changes in lipid components and their relationship with AD still remain inadequately understood. The central nervous system (CNS) is rich in lipoprotein components, particularly apolipoprotein-E (ApoE)-rich high-density lipoprotein (HDL) (Vance and Hayashi, 2010), and the ApoE polymorphism has been stablished as a major risk factor for AD (Corder et al., 1993). In addition to cholesterol, ApoE types can affect the metabolism of sphingolipids. Actually, emerging evidence suggests that sphingolipids play important roles in the pathogenesis of AD, starting from earlier stages of the disease (Kosicek et al., 2012). For example, sphingolipids play structural roles in cellular membranes, including lipid rafts, which imply their involvement in Aβ metabolism (Olsen and Faergeman, 2017).

The brain expresses various sphingolipids, and multiple studies have examined the changes in sphingolipid levels in patients with AD. Among them, sphingosine 1-phosphate (S1P) and ceramides (Cer) have been extensively studied in the field of neurology (Ceccom et al., 2014; Couttas et al., 2014; Ghasemi et al., 2016). Sphingolipids can interconvert among themselves, forming a dynamic and intricate metabolic map. For instance, S1P is produced from sphingosine, and ceramides are derived from sphingomyelins, which can also be converted into sphingosine. Moreover, dihydrosphingosine 1-phosphate (dhS1P) is another ligand for S1P receptors that is produced from dihydrosphingosine (dhSph). The levels of sphingomyelins (SMs) have been reported to be elevated in AD brains (Kosicek et al., 2012; Barbash et al., 2017), whereas S1P levels are decreased (He et al., 2010; Couttas et al., 2014). However, the findings pertaining to ceramides are conflicting, with some studies reporting increased levels (Satoi et al., 2005) and one study reporting decreased levels in AD brains (Barbash et al., 2017). Because sphingolipids form an intricate metabolic map; and not only S1P and Cer, but also other sphingolipids [such as ceramide 1-phosphate (Cer1P) and deoxy-ceramide (Deoxy-Cer)] can be involved in the pathogenesis of AD, considering their potential biological properties (Bertea et al., 2010; Othman et al., 2012; Presa et al., 2020), a comprehensive analysis of sphingolipids is necessary to understand their modulation and estimate the potential roles of the disturbed metabolism of sphingolipids in AD.

Therefore, in the current study, we aimed to obtain a deeper understanding of the role of sphingolipids in AD by employing an advanced analytical method. We recently developed a novel LC-MS/MS method (Uranbileg et al., 2024) that allowed us to analyze a broader range of sphingolipid species using consistent solvent, column, and measurement conditions. This innovation expanded our capacity to explore the dynamic changes in sphingolipid metabolism in pathological conditions.

In this study, we utilized this advanced method to comprehensively analyze sphingolipids in both human postmortem brain tissue samples and AD induced pluripotent stem (iPS) cells. Our aim was to obtain insights into how these sphingolipids are modulated and involved in AD. To understand our findings better, we integrated our sphingolipid data with statistical methods based on machine learning techniques, which enabled us to pinpoint the factors that are most closely associated with AD.

## 2 Materials and methods

### 2.1 Tissue samples

Sphingolipids and ceramides were measured in the following samples: human postmortem brain tissues (cerebral cortex) obtained from subjects who had no medical history of AD or showed no evidence of other CNS disorders at the Tokyo Metropolitan Geriatric Medical Center (Braak stage 1–2; *n* = 6); human postmortem brain tissues obtained from subjects with Cerad-B (classified as an intermediate probability of AD, Braak stage 1–2; *n* = 7) (Murayama and Saito, 2004); and human postmortem brain tissues obtained from subjects with AD (Braak stage 5; *n* = 6), representing an advanced pathology related to end-stage disease. All specimens collected from autopsies were obtained from the Brain Bank for Aging Research.^1^ The Aβ contents were measured using a Human β Amyloid (1–40) and (1–42) ELISA kits (298-64601 and 298–62401, WAKO Pure Chemical Industries, Osaka, Japan), and adjusted to the brain protein levels. The characteristics of these patients are listed in Table 1.

**TABLE 1:** Patients characteristics.

	Control	Cerad-B	AD
*N*	6	7	6
Age (mean, SD)	83.17 (3.34)	85.43 (3.33)	86.17 (1.07)
Gender (M/F)	4/2	2/5	3/3
SP (SD)	0.67 (0.47)	1.57 (0.49)	3.00 (0.00)
Braak (SD)	1.33 (0.47)	1.57 (0.49)	5.00 (0.00)
Aβ (pmol/L, SD)	0.636 (0.171)	1.579 (0.380)	1.591 (0.242)
PMI (min, SD)	377 (306)	825 (278)	702 (346)
RIN (SD)	7.9 (0.74)	8.3 (0.40)	8.1 (0.31)

SP, senile plaque; Aβ, amyloid beta; PMI, postmortem interval; RIN, RNA integrity number.

This study was conducted in accordance with the ethical guidelines outlined in the Declaration of Helsinki. Written informed consent was obtained in advance from the brain donors and/or the next of kin. The study design was approved by the Tokyo Metropolitan Geriatric Medical Center and The University of Tokyo Medical Research Center Ethics Committee (2018088NI).

### 2.2 Cell culture

Human AD-patient-derived iPS cells (iPS cell derived neuronal progenitor cells; ReproNeuro AD-patient 1, RCDN003P); PS [a genetically modified variant (P117L) of Presenilin 1, which is one of the causative molecules in AD], introduced into induced pluripotent stem (iPS) cells derived from healthy individuals; and ReproNeuro AD-mutation (RCDN002N) and Control (normal model neurons, RCDN001N) cells were purchased from ReproCELL, Inc. (Tokyo, Japan) and cultured according to the manufacturer’s protocol.

### 2.3 Sample preparation

For the measurement of all sphingolipids, a total of 10 μl of homogenized tissue samples and cell lysates samples were mixed with 10 μl of internal standards [SPLASH LipidoMix™, Cer1P-d7, hexosyl ceramide (HexCer), C_17_S1P, C_17_dhS1P, C1 7:1 Sphingosine (Sph), C17:1 dihydrosphingosine (dhSph), d18:1/17:0 Ceramide (Avanti Polar Lipids)] at 1 ng/ml (final concentration), and the sphingolipid content was extracted with 100 μl of 0.1% formic acid in methanol (Wako Pure Chemical Industries) and 80 μl of acetonitrile (Wako Pure Chemical Industries). The mixtures were sonicated for 5 min and then centrifuged at 16,400 × *g* for 10 min at 4°C. The supernatants were then analyzed using the LC-MS/MS method.

### 2.4 LC-MS/MS analysis

Sphingolipids were measured using the LC-MS/MS system (WATERS Corporation). Briefly, 5.0-μl samples were injected and LC separation was performed using a normal-phase column [InertSustain Amide 3-μm column: 2.1 × 100 mm (UP)] with a gradient elution of solvent A (10 mM ammonium acetate, 95% acetonitrile, and 5% water) and solvent B (10 mM ammonium acetate, 50% acetonitrile) at 0.6 ml/min. The conditions used in this experiment were as follows: a gradient run was performed at 99% solvent A and 1% solvent B for 1 min, followed by a run at 90% solvent A and 10% solvent B for 1 min, a run at 60% solvent A and 40% solvent B for 3 min, and a run at 20% solvent A and 80% solvent B for 2 min. The total run time was 11 min, the target column temperature was 50°C, and the target sample temperature was 10°C.

The mass spectrometer was operated in electrospray ionization-positive ion mode using the following analytical conditions: the cone gas flow was set at 50 L/h, the desolvation gas flow was set at 1,000 L/h, the source temperature was set at 150°C, and the desolvation temperature was set at 600°C.

The analyses were performed in the multiple reaction monitoring mode in the positive ion mode for sphingolipids. The data were analyzed by MassLynx, TargetLynx XS (WATERS Corporation). The monitored lipids are described in Supplementary Table 1. Both the intra-day and inter-day coefficients of variation for this assay were below 20%.

Using this newly established LC-MS/MS method, we conducted an analysis of sphingolipids and their species, including S1P, Sph, dhS1P, dhSph, Cer (24 species), Cer1P (12 species), HexCer (23 species), lactosyl-ceramide (LacCer, 5 species), dh-ceramide (9 species), deoxy-ceramide (9 species), deoxy-dh-ceramide (9 species), and SM (9 species).

After LC-MS/MS measurement, the areas of each sphingolipid are utilized in the subsequent analysis to determine their respective levels. The protein levels of each sample, which were determined by a colorimetric assay for protein concentration (DC protein assay, 500-0116, Bio-Rad Laboratories, Inc. Hercules, CA, USA), and the areas of the corresponding internal standards were then employed to calculate the final concentrations of the measured sphingolipids.

### 2.5 Statistical analysis

Data processing and analysis were performed using the SPSS (Chicago, IL, USA), MetaboAnalyst 5.0^2^ and GraphPad Prism 8.0 software (GraphPad Software, San Diego, CA, USA). To examine the statistical significance of the differences detected in the sphingolipid levels and among the control brains, Cerad-B brains, and AD brains or cells, we used a one-way analysis of variance test or multiple comparisons with Tukey correction using SPSS. The results pertaining to the lipid levels are expressed as the mean and SD. To assess correlations, Spearman’s rank analysis was employed to examine the association between sphingolipids and other components using SPSS. A sparse Partial Least Square-Discriminant Analysis (PLS-DA) or machine learning study was performed using MetaboAnalyst or by SPSS Modeler, respectively, to explore the characteristics of the three diagnostic groups.

Variable Importance in the Projection (VIP) is a metric that was used to quantify the weighted contribution of sphingolipids to the differences between the groups. VIP was calculated as the sum of squares of the PLS loadings, considering the amount of variation explained in each dimension (for additional details, see text footnote 2). The Kendall rank correlation was employed to investigate the association of sphingolipids or clinical (pathological) data [Aβ levels, Braak stage, and senile plaque (SP) grade] with different groups, considering age and gender as covariates of interest.

Significance was set at *p* < 0.05.

## 3 Results

### 3.1 Overall modulation of sphingolipids in AD brains

**FIGURE 1:**
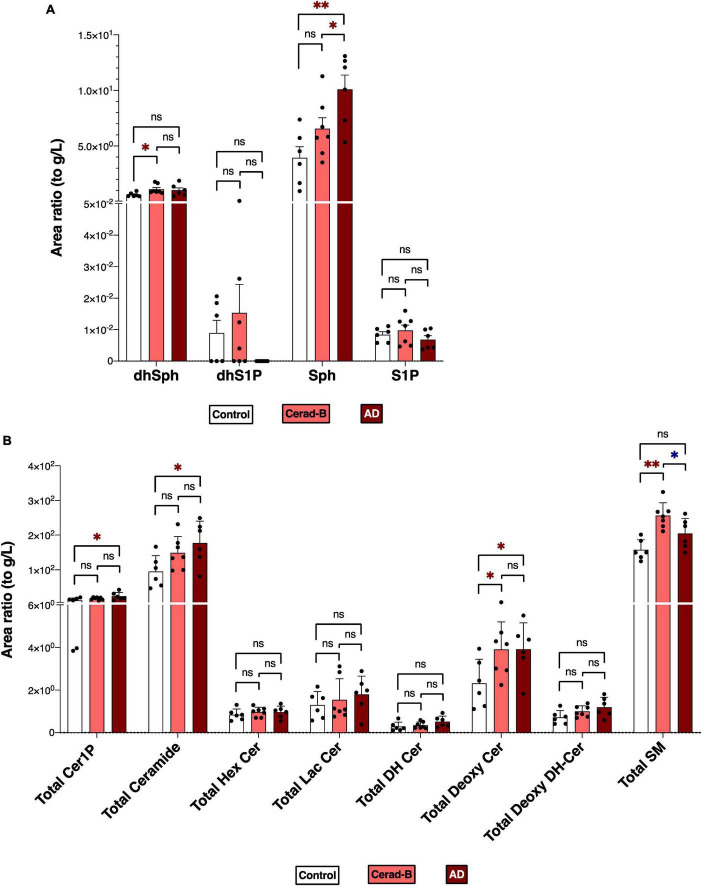
Sphingolipid modulation in AD brain tissues. Sphingolipid levels were assessed in the brains of control subjects (*n* = 6), the patients with Cerad-b (*n* = 7), and those with AD (*n* = 6). Statistical evaluation of differences was conducted using one-way analysis of variance (ANOVA) and multiple comparisons with Tukey correction. **(A)** Dihydrosphingosine (dhSph), dihydrosphingosine 1-phosphate (dhS1P), sphingosine (Sph), and sphingosine 1-phosphate (S1P) levels. **(B)** Total levels of the ceramide 1-phosphate (Cer1P), ceramide (Cer), hexosyl ceramide (HexCer), lactosyl-ceramide (LacCer), dihydroceramide (DH Cer), deoxy-ceramide (Deoxy-Cer), deoxy dihydroceramide (Deoxy-dhCer), and sphingomyelin (SM). Statistical significance was denoted as follows: * for *p* < 0.05, ** for *p* < 0.01, *** for *p* < 0.001. Red * indicates a significant increase, while blue * indicates a significant decrease.

Figures 1A, B reports the total levels of all measured sphingolipids in the three groups. The levels of Sph, total Cer1P, and total Cer were significantly higher in the AD brains compared with the control or Cerad-B brains. The total level of Deoxy-Cer was higher in both the Cerad-B and AD brains compared with the control brains. The levels of dhSph and SM were increased exclusively in the Cerad-B brains, and the levels of S1P were not significantly different among the groups (Figure 1A). The differences in the levels of sphingolipid species with significant changes among the three groups are shown in Supplementary Figures 1A–D.

### 3.2 Modulation of sphingolipid level changes in AD cells

Taking into account that Presenilin is one of the causative genes for familial AD, showing altered Aβ processing and impaired synaptic function, which are characteristics of AD, and considering potential postmortem effects on tissue sphingolipid levels, a similar measurement was conducted on cell lysates from the following three groups (each comprising *n* = 8): iPS cells derived from AD patients (AD), iPS cells with inserted mutations in Presenilin 1 (PS), and control neurons.

**FIGURE 2:**
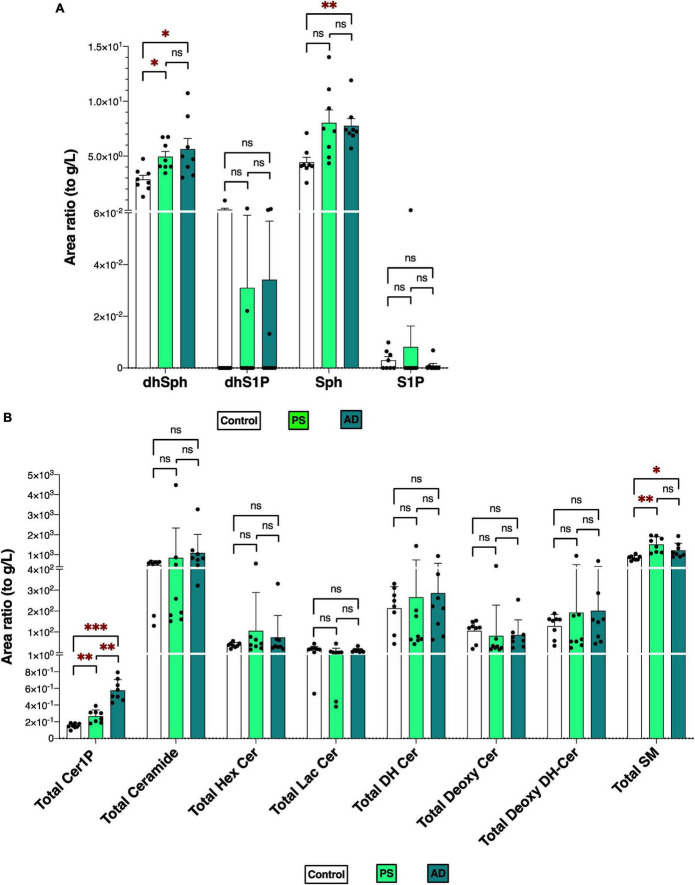
Sphingolipid modulation in iPS cells. Sphingolipid levels were measured in normal model neurons (C), mutated Presenilin 1-inserted iPS cell (PS), and AD patient derived iPS cell (AD), each group *N* = 8. Statistical evaluation of differences was conducted using one-way analysis of variance (ANOVA) and multiple comparisons with Tukey correction. **(A)** DhSph, dhS1P, Sph, and S1P levels. **(B)** Total levels of the Cer1P, Cer, Hex Cer, LacCer, dhCer, Deoxy-Cer, Deoxy-dhCer, and SM. Statistical significance was denoted as follows: * for *p* < 0.05, ** for *p* < 0.01, *** for *p* < 0.001. Red * indicates a significant increase.

Figures 2A, B depicts the total levels of all measured sphingolipid species among the three groups. The dhSph, total Cer1P, and total SM levels were higher in both the PS and AD cells compared with the control cell. The Sph level was enhanced in both the PS and AD cell lines, although statistical significance observed in AD cells exclusively. The levels of S1P were not different among the groups (Figure 2A). Significant differences in the levels of sphingolipid species among the three groups are shown in Supplementary Figures 2A, B.

The comparable modulations observed in Sph and Cer1P levels between the two experiments (Figures 1, 2) offer significant insights into the possible contributions of these sphingolipids to the pathogenesis of AD.

### 3.3 Multivariate data analysis of the sphingolipids

**FIGURE 3:**
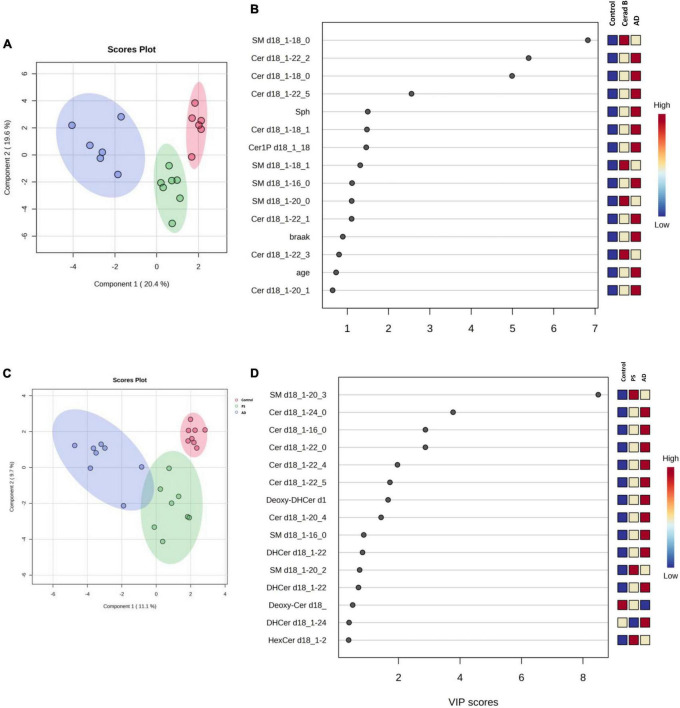
Multivariate analysis of sphingolipids in AD brain tissues and cells. Score plots illustrate distinct profiles of sphingolipids among AD brains **(A)**, Cerad-B brains, and control brains, as well as among AD, PS, and control cells **(C)**. Variable Importance in the Projection (VIP) plots, derived from the PLS-DA models, ranked sphingolipids according to their discriminative ability in brain tissue **(B)** and cell samples **(D)**.

The differential profiles of sphingolipids between AD, Cerad-B, and control brains were investigated using a PLS-DA. The score plots, as depicted in Figure 3A, revealed a significant difference among these three groups. A similar notable difference was observed in the cell samples, as illustrated in Figure 3C.

VIP plots for component 1, which separated the three groups well, were generated from the PLS-DA models, and sphingolipids were ranked based on their discriminatory power for distinguishing the three groups in both brain tissue (Figure 3B) and cell (Figure 3D) samples. Notably, several Cer species exhibited high VIP scores in both the brain tissue and cell samples. Subsequently, the SM species were ranked as significant, although they showed elevated levels in the Cerad-B group (SM 18:0) in the tissue samples and in the PS group (SM 20:3) in the cell samples. Interestingly, the sphingolipid profiles obtained in postmortem brain tissues exhibited a stronger discriminatory power than did the Braak score, which is a measure used to describe the progression of AD, as shown in Figure 3B.

**FIGURE 4:**
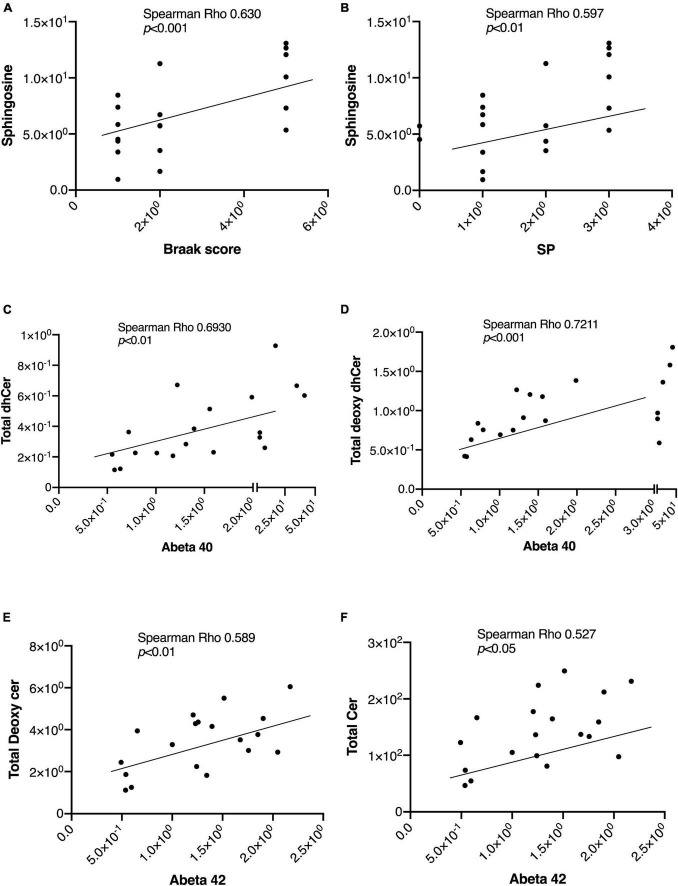
Representative correlations between sphingolipids and the AD-associated markers. The correlations between sphingolipids and AD-associated markers were assessed using Spearman rank correlation. **(A,B)** Sph correlation with the Braak score and SP. **(C,D)** Correlations of Aβ40 levels with total Deoxy-dhCer and total dhCer. **(E,F)** Correlations of Aβ42 levels with total Deoxy-Cer and total Cer.

In Table 2, we present an overview of the correlations between patient characteristics and the measured sphingolipids. Notably, Spearman’s Rho analysis revealed strong correlations between Sph and the Braak score or SP (Figures 4A, B). Additionally, we observed significant correlations between Aβ levels as follows: Aβ40 with total dhCer or total Deoxy-dhCer (Figures 4C, D), and Aβ42 with total Deoxy-Cer or total Cer (Figures 4E, F). The levels of measured Aβ40, Aβ42, and their ratio are provided in Supplementary Table 2. Aβ40 exhibited strong correlations with parameters such as SP and Braak scores, compared to Aβ42. Furthermore, it showed strong correlations with dhCer or total Deoxy-dhCer, in addition to correlations similar to Aβ42.

**TABLE 2:** Correlation between sphingolipids and patient characteristics.

		Sph	SP	Braak	Age	Abeta 40	Abeta 42	dhSph	Total HexCer	Total LacCer	Total dhCer	Total Deoxy-Cer	Total Cer	Total Deoxy-dhCer	Gender	dhS1P	S1P
Total Cer1P	Rho coefficient	0.4884	0.3950	0.4719	0.2783	0.5228	0.2754	0.0885	0.1043	0.3957	0.3331	0.3194	0.4755	0.4382	−0.0072	−0.3394	−0.1978
	*p*-Value	0.0339	0.0941	0.0414	0.2486	0.0216	0.2538	0.7186	0.6708	0.0935	0.1635	0.1825	0.0396	0.0606	0.9767	0.1551	0.4170
Sph	Rho coefficient		0.5966	0.6302	−0.0954	0.5947	0.3749	0.4545	0.2847	0.0884	0.0932	0.3172	0.3588	0.2941	−0.0669	0.1520	−0.1758
	*p*-Value		0.0070	0.0038	0.6976	0.0072	0.1138	0.0506	0.2374	0.7189	0.7044	0.1857	0.1314	0.2217	0.7855	0.5345	0.4717
SP	Rho coefficient			0.8670	0.2838	0.7998	0.5723	0.2023	0.0100	0.2343	0.4544	0.3573	0.4572	0.4669	0.0666	−0.1351	−0.0928
	*p*-Value			0.0000	0.2391	0.0000	0.0104	0.4063	0.9677	0.3344	0.0506	0.1332	0.0491	0.0439	0.7865	0.5814	0.7056
Braak	Rho coefficient				0.3076	0.6892	0.4425	0.0873	0.0254	0.0865	0.3934	0.2113	0.3728	0.3582	0.0753	−0.3142	−0.2523
	*p*-Value				0.2002	0.0010	0.0578	0.7223	0.9177	0.7247	0.0956	0.3852	0.1160	0.1321	0.7595	0.1903	0.2973
Age	Rho coefficient					0.3958	0.5096	0.1492	0.1968	−0.0184	0.3959	0.3576	0.3769	0.3541	0.1888	−0.3858	−0.0960
	*p*-Value					0.0935	0.0258	0.5420	0.4195	0.9404	0.0934	0.1328	0.1117	0.1369	0.4390	0.1028	0.6957
Abeta 40	Rho coefficient						0.6719	0.4509	0.3246	0.4123	0.6930	0.6649	0.7544	0.7211	−0.0193	−0.4692	−0.2737
	*p*-Value						0.0016	0.0527	0.1752	0.0794	0.0010	0.0019	0.0002	0.0005	0.9377	0.0427	0.2357
Abeta 42	Rho coefficient							0.4776	0.3443	0.2767	0.3679	0.5887	0.5274	0.4204	0.1915	−0.2497	−0.1001
	*p*-Value							0.0386	0.1489	0.2515	0.1212	0.0080	0.0203	0.0731	0.4323	0.3026	0.6834
dhSph	Rho coefficient								0.5045	0.2407	0.2016	0.5729	0.4937	0.4150	−0.2045	0.0862	−0.1697
	*p*-Value								0.0276	0.3210	0.4079	0.0103	0.0317	0.0772	0.4011	0.7258	0.4875
Total HexCer	Rho coefficient									0.3281	0.3214	0.6073	0.5525	0.4186	−0.2941	−0.0067	−0.0421
	*p*-Value									0.1702	0.1797	0.0058	0.0142	0.0745	0.2217	0.9783	0.8642
Total LacCer	Rho coefficient										0.6086	0.6233	0.6738	0.6618	−0.3416	−0.2241	0.0793
	*p*-Value										0.0057	0.0044	0.0016	0.0020	0.1524	0.3564	0.7469
Total dhCer	Rho coefficient											0.7615	0.8446	0.8468	−0.4141	−0.0751	−0.1302
	*p*-Value											0.0002	0.0000	0.0000	0.0780	0.7600	0.5953
Total Deoxy-Cer	Rho coefficient												0.9395	0.9029	−0.1635	0.0534	−0.1773
	*p*-Value												0.0000	0.0000	0.5037	0.8280	0.4678
Total Cer	Rho coefficient													0.9748	−0.2670	−0.0952	−0.1526
	*p*-Value													0.0000	0.2691	0.6982	0.5328
Total Deoxy-dhCer	Rho coefficient														−0.2171	−0.0811	−0.1516
	*p*-Value														0.3720	0.7415	0.5357
Gender	Rho coefficient															−0.1911	−0.0442
	*p*-Value															0.4332	0.8573
dhS1P	Rho coefficient																0.2447
	*p*-Value																0.3126

Cer1P, ceramide 1-phosphate; Sph, sphingosine; SP, senile plaque; Abeta, amyloid beta; dhSph, dihydrosphingosine; HexCer, hexosyl ceramide; LacCer, lactosyl-ceramide; dhCer, dihydroceramide; Deoxy-Cer, deoxy-ceramide; Cer, ceramide; Deoxy-dhCer, deoxy dihydroceramide; dhS1P, dihydrosphingosine 1-phosphate; S1P, sphingosine 1-phosphate. The background color dimension indicates the correlation rate, with a stronger correlation represented by the darkest color.

### 3.4 Prediction model identified ceramides and sphingomyelins as key sphingolipids in AD

**FIGURE 5:**
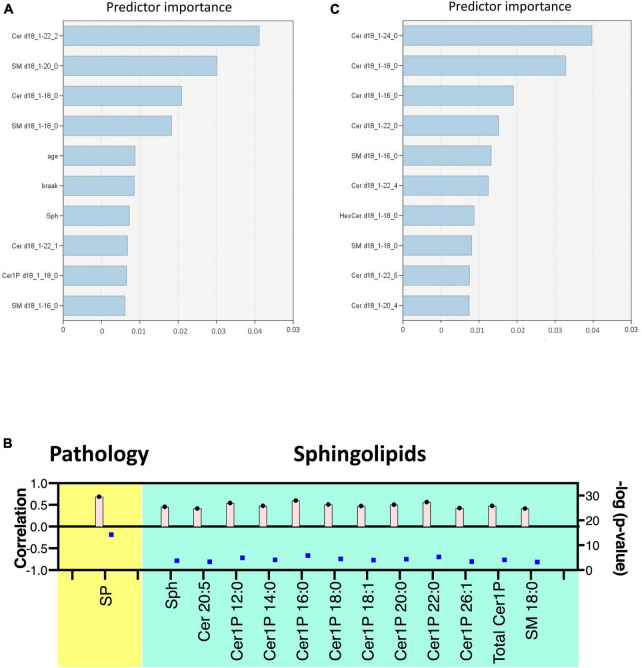
Discriminant analysis using the machine learning model. Illustration of predictor importance in brain tissue **(A)** and cell samples **(C)** using SPSS Modeler’s Predictor Importance Chart in the SVM model. This chart delineates the relative importance of each predictor, highlighting the top 10 sphingolipids ranked by their importance. **(B)** Association of measured sphingolipids with study groups was examined using the Kendall rank correlation. The analysis considered age, gender, and clinical (pathological) data [Aβ levels, Braak stage, and senile plaque (SP) grade] as covariates of interest.

Lastly, we constructed machine learning models for discriminating the groups using IBM SPSS Modeler, which recommended three different models to identify the most relevant sphingolipids associated with AD. The diagnostic performance of these selected machine learning models, together with the most important sphingolipids, exhibited an excellent predictive performance, with nearly 100% accuracy (Supplementary Table 3). As illustrated in Figure 5A, the predictor importance analysis identified specific sphingolipids, including Cer 22:2, SM 20:0, Cer 18:0, and SM 18:0, as having a higher value than the Braak score in postmortem brain tissue samples. Furthermore, we conducted additional correlation analyses, incorporating patients’ age, gender, Aβ levels, Braak stage, and SP grade as covariates of interest with groups (Figure 5B). Notably, SP showed a robust positive association with AD among clinical and pathological data. In relation to sphingolipids, Sph, several species as well as total Cer1P, and certain species of Cer and SM, demonstrated positive associations with AD.

For cell samples, the most important predictors included Cer 24:0, Cer 18:0, Cer 16:0, Cer 22:0, and SM 16:0 (Figure 5C). The assessment of predictor importance was conducted using the Support Vector Machine model, which is an effective and flexible classification method. Across all recommended models, Deoxy-Cer and its species emerged as the most crucial sphingolipids, ranking just below Aβ in importance (Supplementary Table 4) in both brain tissue samples and cell samples.

This application of machine learning models highlighted sphingolipids, including Deoxy-Cer, Sph, several species of Cer, and SM, as key contributors to AD, and suggested their potential for application in AD diagnosis and research.

## 4 Discussion

**FIGURE 6:**
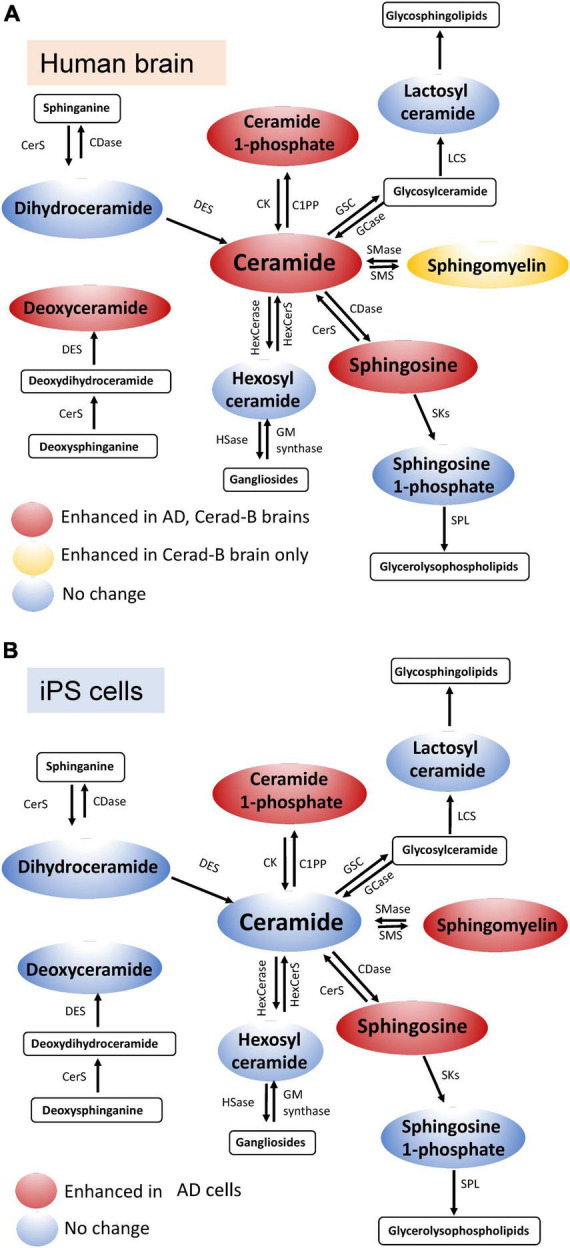
Schematic illustration of sphingolipid modulation in AD. Graphs depicting the modulation of sphingolipids in brain tissue **(A)** and cell samples **(B)** relevant to AD.

In the current study, we used our newly established method (Uranbileg et al., 2024) in combination with multivariate analysis and machine learning models to identify Sph, Cer1P, Cer, and SM as possible candidates for involvement in the pathogenesis of AD. Considering Presenilin as one of the causative genes for familial AD, and acknowledging the potential influence of postmortem effects and background pathological conditions of the patients, such as type 2 diabetes mellitus, on sphingolipid modulation, we also investigated the modulation of sphingolipids using iPS cells as one of models of AD. We obtained similar results in the latter analysis, which helped us conclude on the significant modulation of sphingolipids, especially Sph and Cer1P, in the pathogenesis of AD (summarized in Figure 6A-tissue, Figure 6B-cell).

Sphingolipids are among the well-studied bioactive lipid mediators, playing crucial roles in the physiology and pathology of human diseases (Kluk and Hla, 2002; Hla, 2003; Nakamura et al., 2021; Kurano et al., 2022a,b,d; Uranbileg et al., 2022). In the field of neurology, their structural role in cellular membranes, particularly in lipid rafts, is a crucial aspect of their interaction with the amyloid beta (Aβ) metabolism (Olsen and Faergeman, 2017), suggesting the potential to investigate their role in AD. Recently, we developed a method that enabled us to measure not only an extended range of sphingolipids, but also numerous species of these sphingolipids simultaneously (Uranbileg et al., 2024; Supplementary Table 1). Sphingolipids can interconvert among themselves, thus forming a dynamic and intricate metabolic network; this method allows us to observe comprehensive dynamic changes in sphingolipid levels.

The total ceramide levels were elevated in the brain tissue of patients with AD and its species were identified as either those with the highest VIP score or one of the top 10 important sphingolipid predictors (Figures 1, 3B) in the AD group. This finding was supported by evidence that indicates the presence of increased Cer (Satoi et al., 2005; Akyol et al., 2021) in the cerebrospinal fluid (CSF) or tissues of patients with AD. Furthermore, the exogenous addition of Cer and an increase in endogenous Cer levels lead to higher levels of Aβ and the progression of AD (Puglielli et al., 2003). The total Cer level was also positively correlated with SP grade and Aβ40, 42 (Figure 4 and Table 2), which suggests its importance in AD. Over the past two decades, Cer has been intensively studied regarding its function in inflammation and apoptosis. Many stimuli, such as inflammatory mediators, UV radiation, and oxidative stress, activate cellular sphingomyelinase (SMase) to produce Cer (Kolesnick and Fuks, 2003; Ogretmen and Hannun, 2004). In turn, Aβ activates SMase to produce Cer from SM, and promotes the intracellular accumulation of Cer, which induces oligodendrocyte death (Lee et al., 2004). Cer accumulation is observable at the very early stages of AD, and even in subjects with mild cognitive impairment, which is a disorder that has been associated with risk of AD (Han et al., 2002), and it levels increase before the clinical recognition stages and are elevated in various brain regions (Katsel et al., 2007). Considering the proapoptotic properties of Cer, it seems reasonable that its levels are higher in AD brains and AD cells, thus promoting the pathogenesis of AD.

In both brain tissues and cell lines from individuals with AD, the Sph levels were significantly increased compared with the remaining two groups (Figures 1, 2). Sph was identified as an important factor in AD based on multivariate analysis and machine learning models, suggesting its potential role in AD (Figures 3B, 5A). In addition, Sph was positively and strongly correlated with the SP grade and Braak score of patients with AD (Figures 4A, B and Table 2), which suggests its importance in the diagnosis and underlying mechanism of AD. In similar studies that employed lipid profiling (Cutler et al., 2004; Akyol et al., 2021), sphingolipids such as Cer, SM, and S1P were predominantly mentioned, with less emphasis placed on Sph. Sph and S1P are interconverted by the actions of sphingosine kinase (SK) and S1P phosphatase (S1PP) (Cuvillier, 2002; Maceyka et al., 2002). Aβ disturbs S1P signaling by promoting the accumulation of the proapoptotic Cer, and downregulates SKs, leading to decreased S1P levels (Gassowska et al., 2014). In our case, the S1P levels remained unchanged in tissue or cell samples, which is consistent with previous studies (He et al., 2010; Ceccom et al., 2014; Couttas et al., 2014; Kurano et al., 2022c), which reported either unchanged or decreased S1P levels and SK activity, suggesting a difficulty to employ S1P receptor agonists. These discrepancies might be attributed to postmortem effects, because S1P can be intracellularly degraded (Uranbileg et al., 2022) or because of the lower accuracy of the comprehensive measuring method in the measurement of S1P versus the specific measuring methods of S1P. In fact, in our previous paper using a specific measuring method of S1P, the brain S1P levels were lower in the AD brains (Kurano et al., 2022c).

Regarding Cer1P, our research was the first to suggest that the total levels of Cer1P were higher in the brains of patient with AD and in PS or AD cells. Similar to Sph, Cer1P exhibited a positive correlation with the Braak score of patients with AD, indicating its potential involvement in AD pathology. Cer1P, which is derived from Cer via the action of ceramide kinase (CerK), is recognized for its role in stimulating cell growth and migration, as well as its association with inflammation and apoptosis (Presa et al., 2020). Notably, previous studies have indicated that Cer1P stimulates cell proliferation and adipogenesis, rather than inflammation and apoptosis. Nevertheless, although its precise involvement in AD remains unclear, our study demonstrated its elevation in AD cases. As a downstream product of Cer, further research is crucial to provide conclusive evidence of the role of Cer1P in AD in the near future.

Compared with significantly altered sphingolipids, the newly measured sphingolipid, Deoxy-Cer, exhibited an increase in brain tissues from individuals with Cerad-B scores and patients with AD compared with the control group (Figure 1B). This suggests its importance, as indicated by machine learning models that ranked it just after Aβ, whether considering its total levels or those of its species (Supplementary Table 4) or its strong correlations with Aβ40 and Aβ42 (Table 2) both in brain tissues and cell lines. The involvement of Deoxy-Cer in hereditary sensory autonomic neuropathy and type 2 diabetes mellitus (DM) has been acknowledged, emphasizing its significance in specific medical conditions (Bertea et al., 2010; Othman et al., 2012). Because of the association between Deoxy-Cer and the neuropathy caused by type 2 DM, its potential involvement in AD might be explained by the close relationship between metabolic syndrome or insulin resistance and the progression of AD (Farooqui et al., 2012). Although the physiological properties of Deoxy-Cer remain largely unknown at present, our study suggested the necessity for investigating its roles in CNS diseases.

Although the patterns of the levels of other sphingolipids, such as dhSph and especially SM, were somewhat distinct, some of those species were selected as important factors for AD in the multivariate analysis and machine learning models. Elevated SM levels have been reported in the CSF of patients with prodromal AD (Kosicek et al., 2012), supporting our findings of notably high SM levels in the Cerad-B group among patients or PS cells. Compared with our previous study, in which we conducted lipid profiling for all bioactive lipids and their related factors (Kurano et al., 2022c), we noticed some discrepancies in the levels of Sph, SM, and dhSph, which could be attributed to the improved methodology.

Because of the progressive nature of AD and the gradual worsening of dementia-related symptoms and pathology over time, the significance of early-stage dementia biomarkers becomes increasingly important. Therefore, here, we employed three groups for each type of sample (control, mild stage; Cerad-B for brain tissue; and AD for advanced stage) to explore the differences in sphingolipid metabolism at various stages of AD. With the exception of Cer1P in cell lines, which exhibited a significant gradual increase, no significant variations in lipid profiles were observed across the different AD stages, excluding a gradually increasing tendency in the levels of the Sph and total Cer.

The ApoE gene, particularly its ε4 allele, is widely recognized as the most influential genetic risk factor for AD, affecting over half of all cases (Raulin et al., 2022). ApoE proteins, encoded by various alleles, primarily facilitate lipid transport while also participating in diverse biological functions. Therefore, we conducted additional analysis based on ApoE gene alleles. Although we observed a tendency toward increased levels of certain sphingolipids in Group 1 (ApoE 3/4), the findings were not statistically significant (Supplementary Figure 3). It is worth noting that there were no patients with the ApoE 4/4 allele in our study group. Although the present study did not show the impact of ApoE allele on sphingolipid metabolism, further research is warranted to thoroughly elucidate the modulation of sphingolipids in response to the ApoE ε4 allele.

The primary limitations of this study included the sample size of each group and the absence of clinical information beyond what is presented here, as well as the possible postmortem effects on metabolites. Although the postmortem interval (PMI) tended to be shorter in the control subjects, no significant difference was observed among the three groups. The conditions of the brain samples seemed not to be significantly different, considering that the RNA integrity number was not so different among the samples (Table 1). The RIN values are higher than 7, indicating the good quality of the brain tissues. It should also be noted that, considering the variability of the tissue sample origin, in this instance, we utilized postmortem cerebral cortex tissues. Therefore, further sphingolipid profiling should encompass analyses of the white matter and hippocampal sections from brain tissues, as well as glial cells in cell line studies, considering that we utilized neurons in the present analysis. Another limitation concerns the iPS cells used in this study, as it is important to note that the iPS cell lines utilized here are characterized by Presenilin mutations. While these mutated lines exhibit certain AD-associated traits (Kim and Tanzi, 1997; Chen et al., 2002; Bagaria et al., 2022), including altered Aβ processing (Chen et al., 2002) and compromised synaptic function (Naruse et al., 1998; Yoo et al., 2000), they do not fully replicate all AD hallmark features. Therefore, future investigations should involve additional AD-specific iPS lines, such as those with MAPT mutations (Strang et al., 2019), and explore the use of more physiologically relevant models like organoids or animal models. These approaches aim to better encompass the diverse spectrum of lipid profiles observed within the brain, providing the possibility to elucidate the main mechanisms of the modulated sphingolipids, whether they are related to certain mutations or to outcomes observed in AD brain tissue.

## 5 Conclusion

In summary, a novel method combined with an advanced data analysis identified key sphingolipids for diagnosing AD, including Sph, Cer, Cer1P, and SMs. These sphingolipids were highlighted as a critical AD marker, as they exhibited strong correlations with AD-related factors and ranked above the Braak score as an important factor. We believe that this study could serve as a foundational pillar for further AD research, particularly for understanding the involvement of bioactive lipids.

## Data availability statement

The original contributions presented in this study are included in this article/Supplementary material, further inquiries can be directed to the corresponding author.

## Ethics statement

The studies involving humans were approved by the Tokyo Metropolitan Geriatric Medical Center and The University of Tokyo Medical Research Center Ethics Committee (2018088NI). The studies were conducted in accordance with the local legislation and institutional requirements. The participants provided their written informed consent to participate in this study.

## Author contributions

BU: Data curation, Formal analysis, Investigation, Visualization, Writing – original draft. HI: Formal analysis, Validation, Writing – review and editing. ES: Methodology, Writing – review and editing. MKb: Methodology, Writing – review and editing. YS: Project administration, Resources, Writing – review and editing. MKr: Conceptualization, Formal analysis, Funding acquisition, Project administration, Validation, Writing – review and editing.

## Footnotes

1
https://www.tmig.or.jp/eresearch/a23.html

2
https://www.metaboanalyst.ca/
