# Supplementary material for: Alzheimer’s disease manifests abnormal sphingolipid metabolism
Source: Front Aging Neurosci. 2024 May 7;16:1368839. doi: 10.3389/fnagi.2024.1368839 (PMC11106446; doi:10.3389/fnagi.2024.1368839)
Supplement: Supplementary file 1 [file Presentation_1.pdf]

Supplementary Table 1. List of monitored sphingolipids

#	Name	#	Name
1	DihydroSphingosine	65	HexCer d18_1-22_3
2	DihydroSphingosine-1- Phosphate	66	HexCer d18_1-22_4
3	IS DihydroSphingosine	67	HexCer d18_1-22_5
4	IS DihydroSphingosine-1- Phosphate	68	HexCer d18_1-22_6
5	IS Sphingosine	69	SM d18_1-12_0
6	IS Sphingosine-1- Phosphate	70	SM d18_1-14_0
7	Sphingosine	71	SM d18_1-14_1
8	Sphingosine-1- Phosphate	72	SM d18_1-16_0
9	IS_Cer d18_1-17_0	73	SM d18_1-16_1
10	Cer1P d18_1_12_0	74	SM d18_1-17_0
11	Cer1P d18_1_14_0	75	SM d18_1-18_0
12	Cer1P d18_1_16_0	76	SM d18_1-18_1
13	Cer1P d18_0_16_0	77	SM d18_1-18_2
14	Cer1P d18_1_18_1	78	SM d18_1-18_3
15	Cer1P d18_1_18_0	79	SM d18_1-18_4
16	Cer1Pd18_1_20_0	80	SM d18_1-20_0
17	Cer1Pd18_1_22_0	81	SM d18_1-20_1
18	Cer1P d18_1_24_1	82	SM d18_1-20_2
19	Cer1P d18_1_24_0	83	SM d18_1-20_3
20	Cer1P d18_1_26_1	84	SM d18_1-20_4
21	Cer1P d18_1_26_0	85	SM d18_1-20_5
22	Cer d18_1-12_0	86	SM d18_1-22_0
23	Cer d18_1-14_0	87	SM d18_1-22_1
24	Cer d18_1-14_1	88	SM d18_1-22_2
25	Cer d18_1-16_0	89	SM d18_1-22_3
26	Cer d18_1-16_1	90	SM d18_1-22_4
27	Cer d18_1-18_0	91	SM d18_1-22_5
28	Cer d18_1-18_1	92	SM d18_1-22_6
29	Cer d18_1-18_2	93	18:1 (d9) SM
30	Cer d18_1-18_3	94	LacCer d18_0-22_0
31	Cer d18_1-18_4	95	LacCer d18_1-16_0
32	Cer d18_1-20_0	96	LacCer d18_1-22_0
33	Cer d18_1-20_1	97	LacCer d18_1-24_0
34	Cer d18_1-20_2	98	LacCer d18_1-24_1
35	Cer d18_1-20_3	99	IS_HexCer-Glu Cer C12
36	Cer d18_1-20_4	100	IS_Ceramide1P-d7(d18:1)C15
37	Cer d18_1-20_5	101	DHCer d18_1-14_0
38	Cer d18_1-22_0	102	DHCer d18_1-16_0
39	Cer d18_1-22_1	103	DHCer d18_1-18_0
40	Cer d18_1-22_2	104	DHCer d18_1-18_1

41	Cer d18_1-22_3	105	DHCer d18_1-20_0
42	Cer d18_1-22_4	106	DHCer d18_1-22_0
43	Cer d18_1-22_5	107	DHCer d18_1-22_1
44	Cer d18_1-22_6	108	DHCer d18_1-24_0
45	Cer d18_1-24_0	109	DHCer d18_1-24_1
46	HexCer d18_1-12_0	110	Deoxy-DHCer d18_1-14_0
47	HexCer d18_1-14_0	111	Deoxy-DHCer d18_1-16_0
48	HexCer d18_1-14_1	112	Deoxy-DHCer d18_1-18_0
49	HexCer d18_1-16_0	113	Deoxy-DHCer d18_1-18_1
50	HexCer d18_1-16_1	114	Deoxy-DHCer d18_1-20_0
51	HexCer d18_1-18_0	115	Deoxy-DHCer d18_1-22_0
52	HexCer d18_1-18_1	116	Deoxy-DHCer d18_1-22_1
53	HexCer d18_1-18_2	117	Deoxy-DHCer d18_1-24_0
54	HexCer d18_1-18_3	118	Deoxy-DHCer d18_1-24_1
55	HexCer d18_1-18_4	119	Deoxy-Cer d18_1-14_0
56	HexCer d18_1-20_0	120	Deoxy-Cer d18_1-16_0
57	HexCer d18_1-20_1	121	Deoxy-Cer d18_1-18_0
58	HexCer d18_1-20_2	122	Deoxy-Cer d18_1-18_1
59	HexCer d18_1-20_3	123	Deoxy-Cer d18_1-20_0
60	HexCer d18_1-20_4	124	Deoxy-Cer d18_1-22_0
61	HexCer d18_1-20_5	125	Deoxy-Cer d18_1-22_1
62	HexCer d18_1-22_0	126	Deoxy-Cer d18_1-24_0
63	HexCer d18_1-22_1	127	Deoxy-Cer d18_1-24_1
64	HexCer d18_1-22_2		

Supplementary Table 2. Amyloid beta peptides and their ratio.

AD	Ab 40	Ab 42	Ab 40/42
a1	8.330435	1.341975	6.207593
a2	41.25046	1.514723	27.233
a3	34.78282	1.25822	27.64446
a4	17.24852	1.903789	9.060098
a5	4.196559	1.675595	2.504519
a6	4.31239	1.850546	2.330334
Average	18.35±16.1	1.59±0.26	12.49667±11.8
**Cerad-b**	**Ab 40**	**Ab 42**	**Ab 40/42**
b1	1.010412	2.048068	0.493349
b2	1.594374	1.757481	0.907193
b3	1.555295	1.20731	1.288231
b4	1.176586	1.242604	0.946871
b5	1.310181	1.231305	1.064059
b6	1.989103	2.171879	0.915844
b7	1.394254	1.395781	0.998906
Average	1.43±0.28	1.57±0.39	0.94±0.14
**Control**	**Ab 40**	**Ab 42**	**Ab 40/42**
c1	1.220725	0.653409	1.86824
c2	0.549597	0.536317	1.02476
c3	0.720657	1.001111	0.719857
c4	0.787946	0.492575	1.599645
c5	0.574582	0.595149	0.965441
c6	0.634133	0.539165	1.176139
Average	0.75±0.25	0.63±0.19	1.23±0.43

Amyloid beta (Ab) unit: pmol/L

Supplementary Table 3. Accuracy of the used machine learning model

Cell

Record summary

Record	Number	Percentage
Inclusion	19	100
Exclusion	0	0
Total	19	100

Brain tissue

Record summary

Record	Number	Percentage
Inclusion	15	100
Exclusion	0	0
Total	15	100

Supplementary Table 4. Deoxy-Cer is recognized as a significant factor across the recommended models.

Cells		Brain tissue	
**Nodes**	**Importance**	**Nodes**	**Importance**
Deoxy-Cer d18_1-14_0	0.0078	Deoxy-DHCer d18_1-24_1	0.0075
Deoxy-Cer d18_1-16_0	0.0078	Total deoxy dhCer	0.0075
Deoxy-Cer d18_1-18_0	0.0078	Deoxy-Cer d18_1-14_0	0.0075
Deoxy-Cer d18_1-18_1	0.0078	Deoxy-Cer d18_1-16_0	0.0075
Deoxy-Cer d18_1-20_0	0.0078	Deoxy-Cer d18_1-18_0	0.0075
Deoxy-Cer d18_1-22_0	0.0078	Deoxy-Cer d18_1-18_1	0.0075
Deoxy-Cer d18_1-22_1	0.0078	Deoxy-Cer d18_1-20_0	0.0075
Deoxy-Cer d18_1-24_0	0.0078	Deoxy-Cer d18_1-22_0	0.0075
Deoxy-Cer d18_1-24_1	0.0078	Deoxy-Cer d18_1-22_1	0.0075
Total Deoxy cer	0.0078	Abeta	0.0088

Supplementary Figure S1.

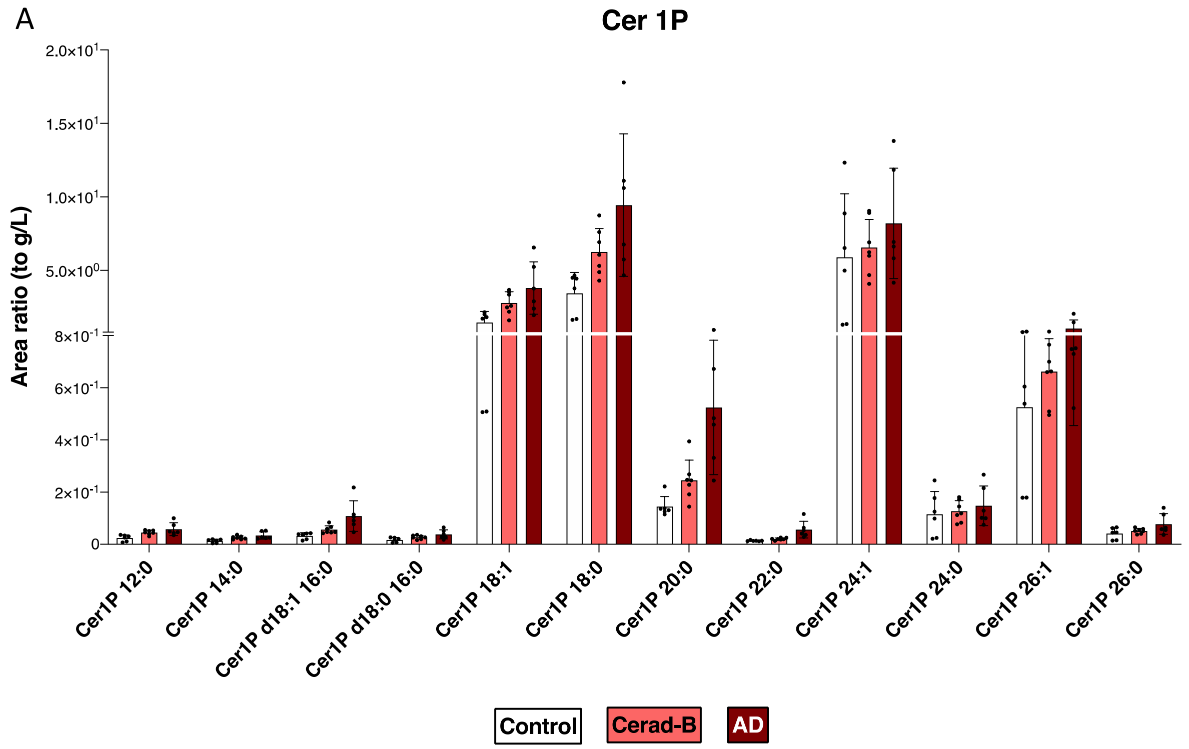

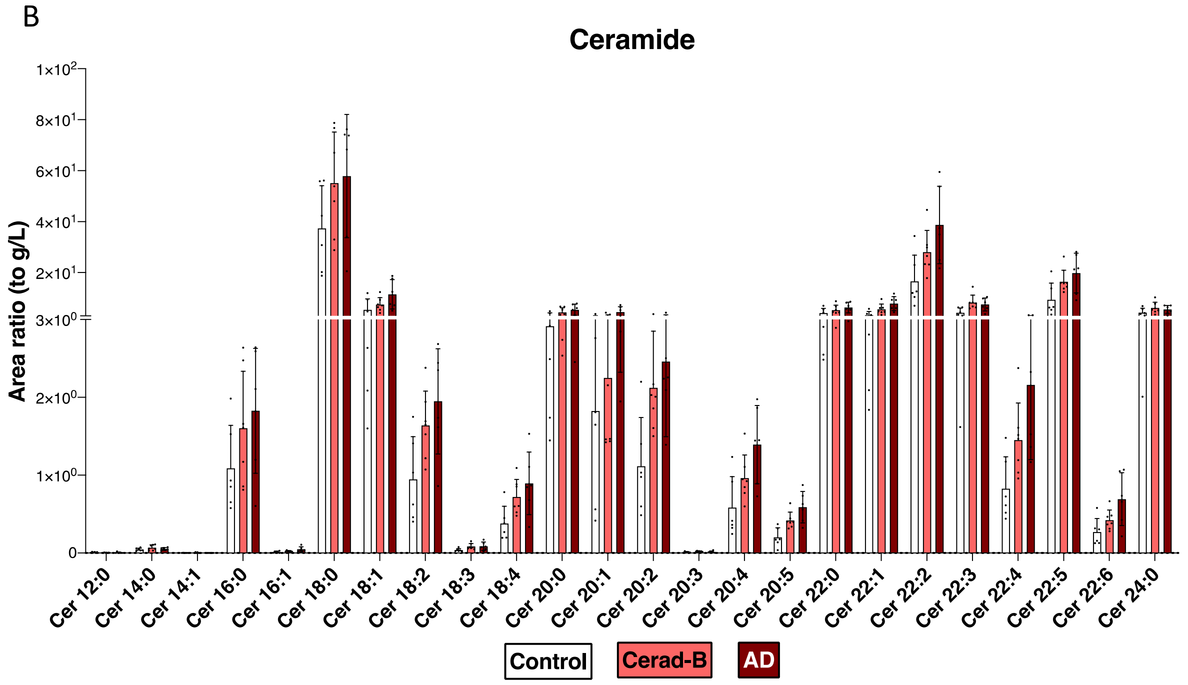

C

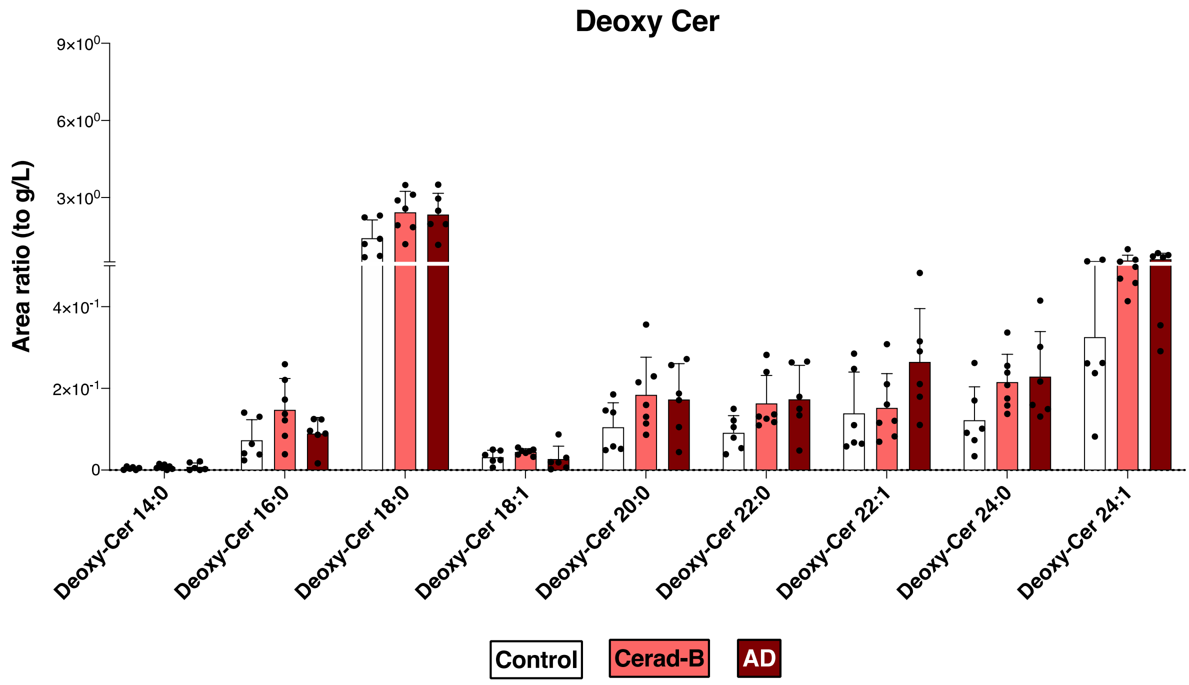

D

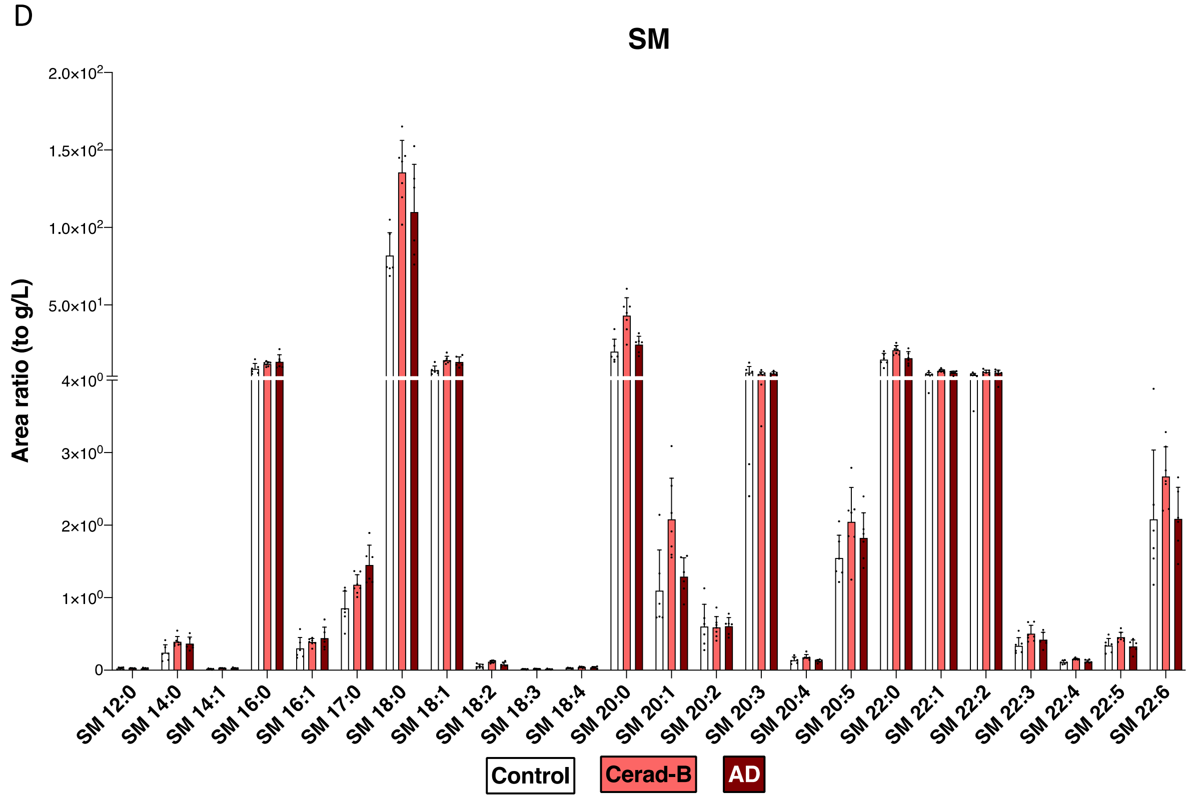

Supplementary Figure S1. Sphingolipid modulation in AD brain tissues.

A-D. Each species of the Cer1P, Cer, Deoxy Cer and SM in the brains of control subjects (*n*= 6), the patients with Cerad-b (*n*= 7), and those with AD (*n*= 6). The results are expressed as the mean ±SD.

Supplementary Figure S2.

A

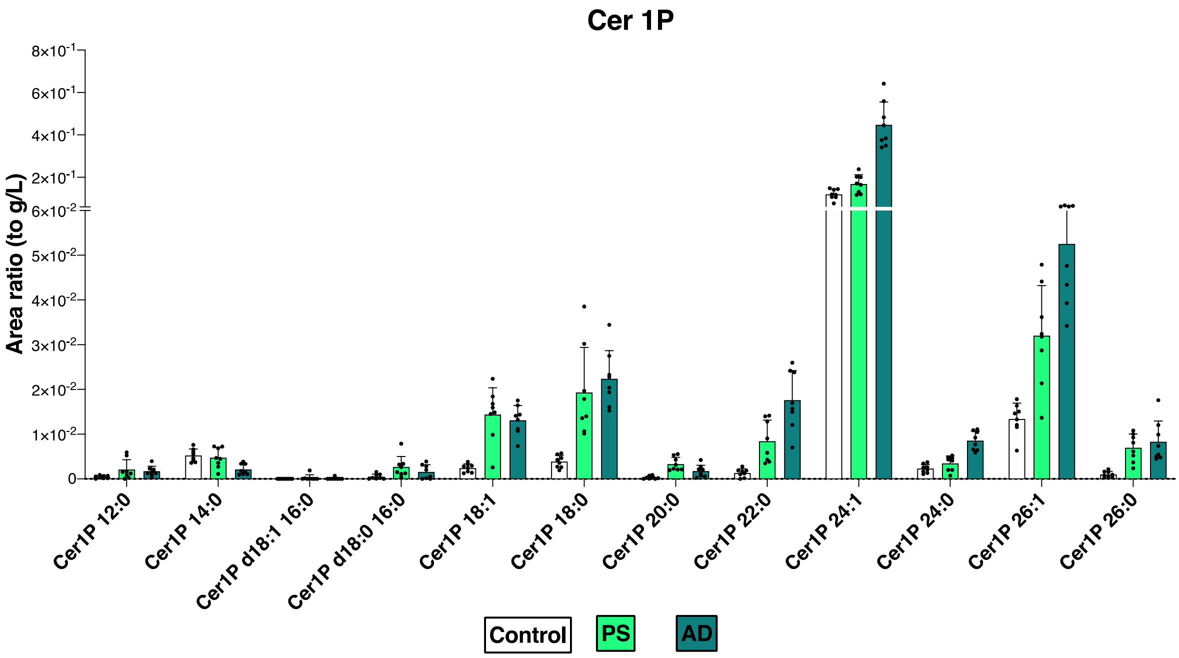

B

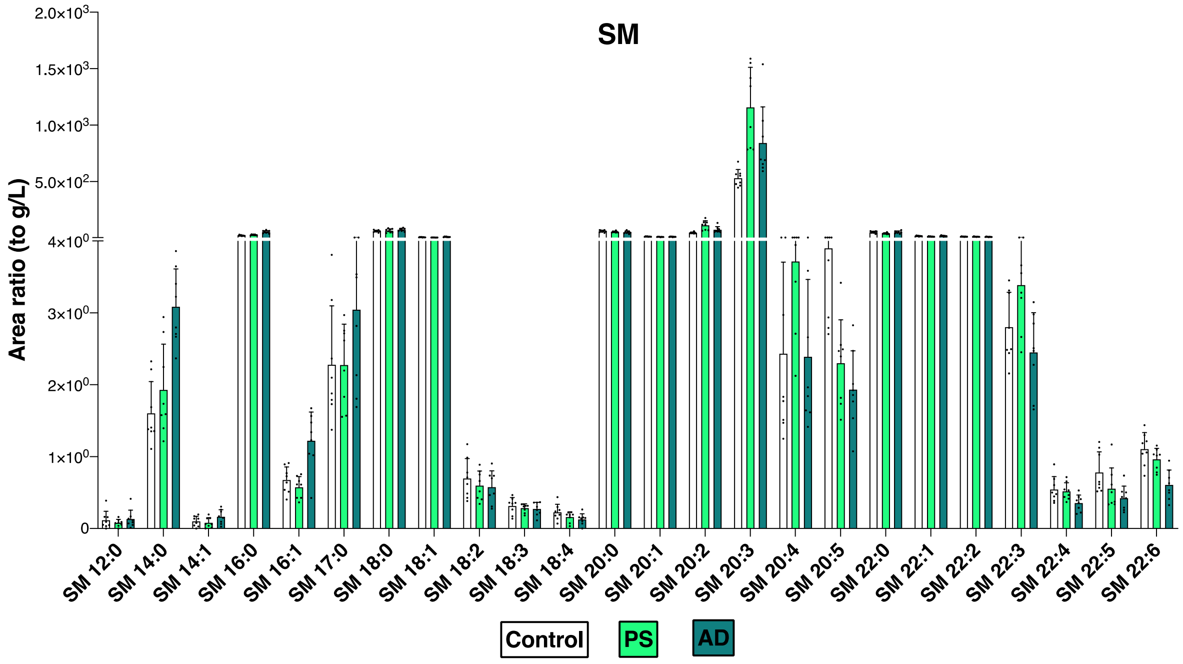

Supplementary Figure S2. Sphingolipid modulation in iPS cells.

A-D. Each species of the Cer1P and SM in normal model neurons (C), mutated Presenilin1-inserted iPS cell (PS), and AD patient derived iPS cell (AD), each group N=8. The results are expressed as the mean ±SD.

Supplementary Figure S3.

A

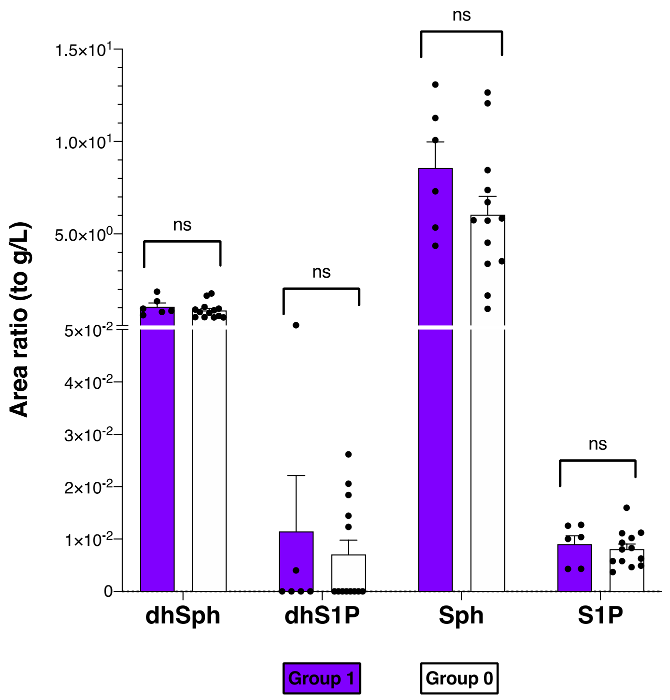

B

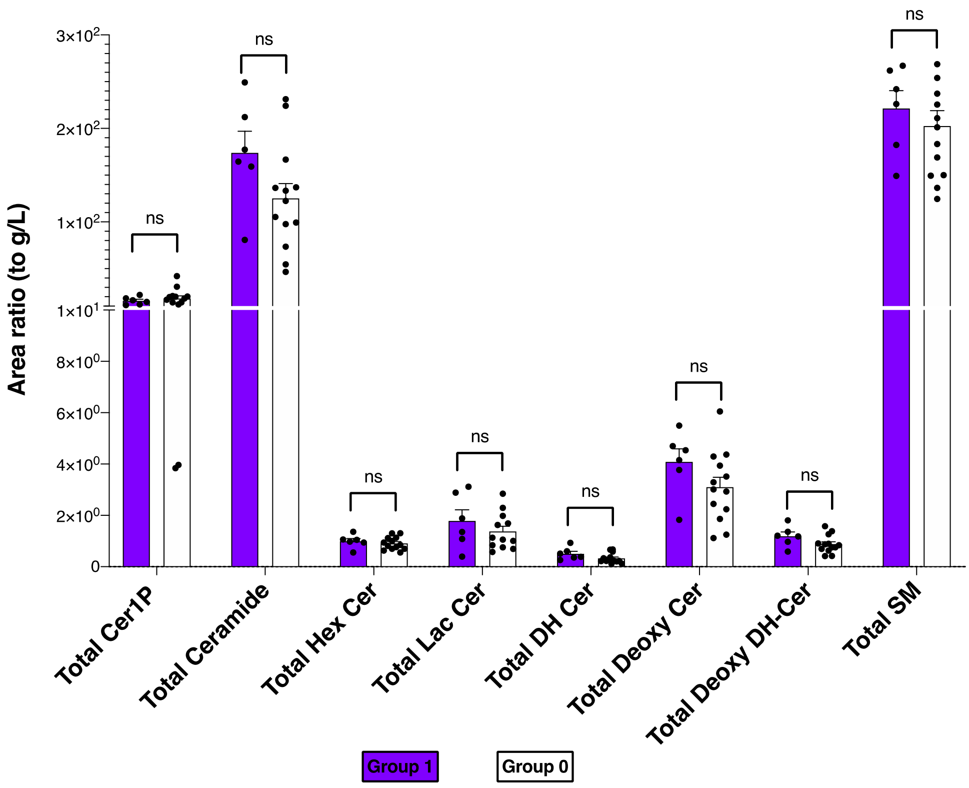

Supplementary Figure S3. Sphingolipid modulation in AD brain tissues based on the ApoE4 allele.

Postmortem brain tissues were categorized based on ApoE allele distribution as follows: Group 1 (purple) included individuals with the 3/4 allele (N=6), while Group 0 (white) comprised individuals with alleles other than 3/4 or 4/4 (N=13). The results are expressed as the mean ±SD.

A. Dihydrosphingosine (dhSph), dihydrosphingosine 1-phosphate (dhS1P), sphingosine (Sph) and sphingosine 1-phosphate (S1P) levels. **B.** Total levels of the ceramide 1-phosphate (Cer1P), ceramide (Cer), hexasyl ceramide (Hex Cer), lactosyl ceramide (Lac Cer), dihydroceramide (DH Cer), deoxy ceramide (Deoxy Cer), deoxy dihydroceramide (Deoxy DH-Cer) and sphingomyelin (SM).
